# Identifying the Intergenic *ALK* Fusion *LOC388942‐ALK* as a Driver of Non–Small Cell Lung Cancer

**DOI:** 10.1002/mco2.70154

**Published:** 2025-03-27

**Authors:** Xiaoqian Zhai, Manli Wang, Qi Zhang, Donglin Li, Yanmou Wu, ZuoYu Liang, Jiewei Liu, Weiya Wang, Yu Liu, Guowei Che, Qinghua Zhou, Chong Chen

**Affiliations:** ^1^ Department of Medical Oncology, State Key Laboratory of Biotherapy and Cancer Center and National Clinical Research Center for Geriatrics West China Hospital Sichuan University Chengdu China; ^2^ Lung Cancer Center West China Hospital Sichuan University Chengdu China; ^3^ State Key Laboratory of Biotherapy and Cancer Center West China Hospital, Sichuan University Chengdu China; ^4^ College of Life Science Sichuan University Chengdu China; ^5^ Department of Pathology of West China Hospital Sichuan University Chengdu China; ^6^ Department of Hematology and Institute of Hematology, State Key Laboratory of Biotherapy West China Hospital, Sichuan University Chengdu China; ^7^ Department of Thoracic Surgery, West China Hospital Sichuan University Chengdu Sichuan China; ^8^ Frontiers Medical Center Tianfu Jincheng Laboratory Chengdu Sichuan China

**Keywords:** *ALK* fusion, *FOS*, intergenic fusion, non–small cell lung cancer, tumorigenesis

## Abstract

*ALK* fusions, such as the classic *EML4‐ALK*, are known drivers of lung cancer and effective therapeutic targets. However, variant *ALK* fusions, including intergenic fusions like *LOC388942‐ALK* (*LA*), have been detected in increasing numbers of patients, with their roles in tumorigenesis and ALK inhibitor resistance remaining unclear. Using CRISPR/Cas9, we generated the *LA* fusion in A549 and H441 cells, confirming elevated ALK expression via qRT‐PCR and immunohistochemistry (IHC) staining. Functional analyses showed that *LA* significantly promoted tumor growth in vitro and in vivo while conferring increased resistance to alectinib. RNA‐seq revealed upregulation of the *FOS* pathway in *LA* tumors, identifying *FOS* as a potential therapeutic target. Subsequently, we demonstrated that FOS disruption and inhibition sensitized *LA* tumors to treatment. RNA‐seq profiling demonstrated that *FOS* depletion in *LOC388942‐ALK* tumor significantly downregulated multiple oncogenic pathways related to cell cycle progression, DNA replication fidelity, and extracellular matrix remodeling, suggesting a pivotal role of *FOS* in maintaining tumor growth. These findings establish *LOC388942‐ALK* as a novel oncogenic driver in lung cancer, highlighting its role in tumor growth and ALK inhibitor resistance. Targeting *FOS* may provide a promising therapeutic strategy for tumors harboring this intergenic fusion.

## Graphical abstract

Intergenic breakpoint fusions are generally nonfunctional due to the lack of chimeric full‐coding transcripts. However, in this study we demonstrate an intergenic fusions *LOC388942‐ALK* (*LA*) drives oncogenesis through accelerated tumor growth (in vitro/vivo) and alectinib resistance. Crucially, FOS inhibition sensitizes *LA* tumors to treatment, establishing *LA* as a targetable NSCLC driver with therapeutic implications for fusion‐positive malignancies.

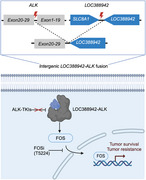

## 1 Introduction

Lung cancer is the leading cause of cancer mortality, posing a significant public health challenge. Non–small cell lung cancer (NSCLC) constitutes 80%–85% of all lung cancers [1]. Anaplastic lymphoma kinase (*ALK*) fusion‐positive lung cancer represents approximately 3%–7% of NSCLC cases [2]. *EML4‐ALK* is the most common *ALK* fusion in NSCLC, accounting for 85% of all ALK‐positive cases [3]. This fusion protein leads to ALK overexpression and activates several downstream pathways, such as PI3K/AKT/mTOR, MAPK/RAS/ERK, and JAK/STAT [4, 5, 6, 7], promoting cancer cell growth. Patients with *EML4‐ALK* are typically treated with ALK tyrosine kinase inhibitors (ALK‐TKIs), significantly improving overall survival to around 35–40 months [8, 9].

However, approximately 15% of *ALK* fusions in NSCLC are considered variant *ALK f*usions involving different fusion partners and mechanisms. Among these, intergenic *ALK* fusions are unique and include breakpoints between two genes that fuse with the *ALK* gene [10]. Intergenic breakpoint fusions are generally nonfunctional due to the lack of chimeric full‐coding transcripts. However, in a previous study, researchers expressed an intergenic fusion, *LIN00308/D21S2088E‐ALK* (L/D‐ALK), in Ba/F3 cells, which requires IL‐3 for growth; the cells grew exponentially even without IL‐3, confirming its oncogenic properties [11]. Furthermore, a recent study showed that intergenic fusions occur more frequently than expected, and their role in tumors may have been overlooked [12]. The study showed that out of 13,698 mutations detected in 268 pan‐cancer samples, 8532 were intergenic fusions, accounting for about 62%, indicating that intergenic fusions may occur in malignant tumors at a much higher frequency than previously estimated [13, 14]. Intergenic *ALK* fusion is likely to become an important target in NSCLC.

The intergenic fusion, *SLC8A1/LOC388942‐ALK* (S/L‐ALK), also called *LOC388942‐ALK* (Lintergenic: A20) fusion, involves a breakpoint between the *SLC8A1* gene and the *LOC388942* intergenic region on chromosome 2, fusing with exons 20–29 of the *ALK* gene [15]. The patient with *LOC388942‐ALK* exhibited clinical features similar to those with *EML4‐ALK*. However, the patient did not respond very well to ALK‐TKIs treatment. Similarly, intergenic fusions, such as *ZIC4/LINC02010‐ALK* (G/C‐ALK) and *ZIC4/LINC02010‐ALK* (Z/L‐ALK), also showed poor responses to ALK‐TKIs treatments [16]. Therefore, studying the tumorigenesis of intergenic *ALK* fusions and discovering new targeted therapies is crucial for improving patients’ survival.

*FOS* is located on chromosome 14q24.32 and is a member of the *FOS* gene family. It is mainly responsible for encoding *c‐FOS*. The c‐FOS protein or other FOS family proteins can form the AP‐1 transcription factor complex together with members of the JUN complex family, thereby binding to the AP‐1 regulatory elements in the gene promoter and enhancer regions to regulate gene expression. Studies have shown that overexpression of FOS can promote tumorigenesis and chemotherapy resistance in tumors such as ovarian cancer and osteosarcoma, and that *c‐FOS* expression is clinically significantly correlated with osteosarcoma recurrence [17]. c‐FOS‐related inhibitors such as T‐5224 have been developed and have been shown to inhibit the metastasis and growth of NSCLC [18].

In this study, we used CRISPR/Cas9 gene editing to generate a spontaneous intergenic fusion cell and mouse model expressing the *LOC388942‐ALK* (*LA*) fusion, demonstrating its oncogenic effect. Using this model, we investigated the molecular mechanisms of *LA* fusion and identified a new susceptible pathway involving *FOS*. Inhibiting this pathway suppressed the growth of *LA* NSCLC cells in vitro and in vivo experiments. These findings provide valuable insights into the mechanisms of intergenic ALK fusion in patients and suggest potential new therapeutic targets for this disease.

## 2 Results

### 2.1 Identifying Clinical Characteristics of Intergenic *ALK* Fusion

**FIGURE 1:**
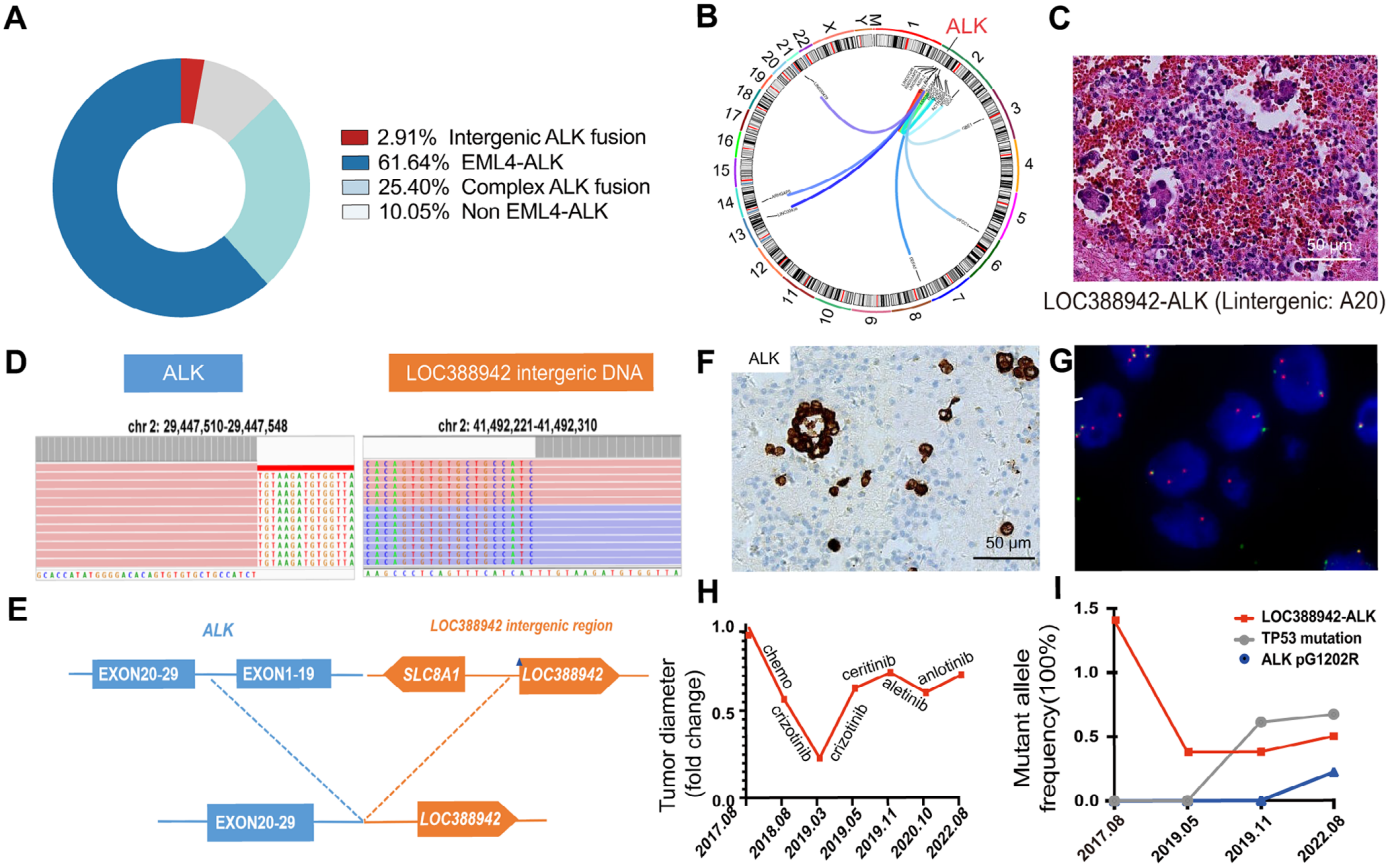
Clinical characteristics of intergenic *ALK* fusion. (A) Pie chart showing the frequency of different subtypes of *ALK* fusion in NSCLC patients. (B) The circular plot showing an overview of the intergenic *ALK* fusion events between locations in chromosomes. (C) Representative H&E staining of tumor with intergenic *ALK* fusion *LOC388942‐ALK* (*LA*). (D) Tumor cell sequencing of the tumor with *LA* fusion. (E) Schematic diagram of *LA* fusion. (F) Representative immunohistochemical staining image of patient with *LA* fusion. (G) Representative ALK FISH diagnosis figure of the patient with *LA* fusion. (H) Tumor diameter fold change of patient with *LA* fusion after chemotherapy and ALK‐TKIs. (I) The frequency of fusion genes and accompanying gene mutations in patients at different stages of disease progression.

We analyzed the collected 378 *ALK* fusion cases, which were detected by immunohistochemistry (IHC) of ALK and next‐generation sequencing and then confirmed by fluorescence in situ hybridization (FISH) of ALK at West China Hospital of Sichuan University. We found tumors with classic *EML4‐ALK f*usion accounted for 61.64%, complex *ALK* fusion gene (*EML4‐ALK* concurrent with other fusions) accounted for 25.40%, the non–*EML4‐ALK* alone fusion gene accounted for 10.05%, and the intergenic *ALK* fusion gene accounted for 2.91% (Figure 1A). Among intergenic *ALK* fusions, most of the fusion is first time reported, such as A*CTR3BP2‐ALK, ASXL2‐ALK* and *LINC01248‐ALK* (Figure 1B). Here, we focused on a previously reported fusion—*LOC388942‐ALK* (*LA*)—as the subject of our study [15]. *LA* fusion without other alternations was identified in a case of advanced lung adenocarcinoma (cT4N0M1a, stage IVa) at diagnosis. The patient exhibited pathological features similar to those of *EML4‐ALK* (Figure 1C). The tumor cell had a break in the intergenic sequence of the *LOC388942* and a rearrangement with the 20–29 exons of the *ALK*. The fusion mechanism was chromosome 2 inversion between p21 and p23.1 [2] (Figure 1D,E). IHC and FISH confirmed the ALK fusion in the patient (Figure 1F,G). The patient was treated with chemotherapy and crizotinib, followed by resistance to ceritinib and alectinib (Figure 1H). *LA* fusion was presented throughout the treatment process (Figure 1I).

### 2.2 Spontaneous Fusion of *LOC388942‐ALK* Was Achieved in A549 Cells and H441 Cells

**FIGURE 2:**
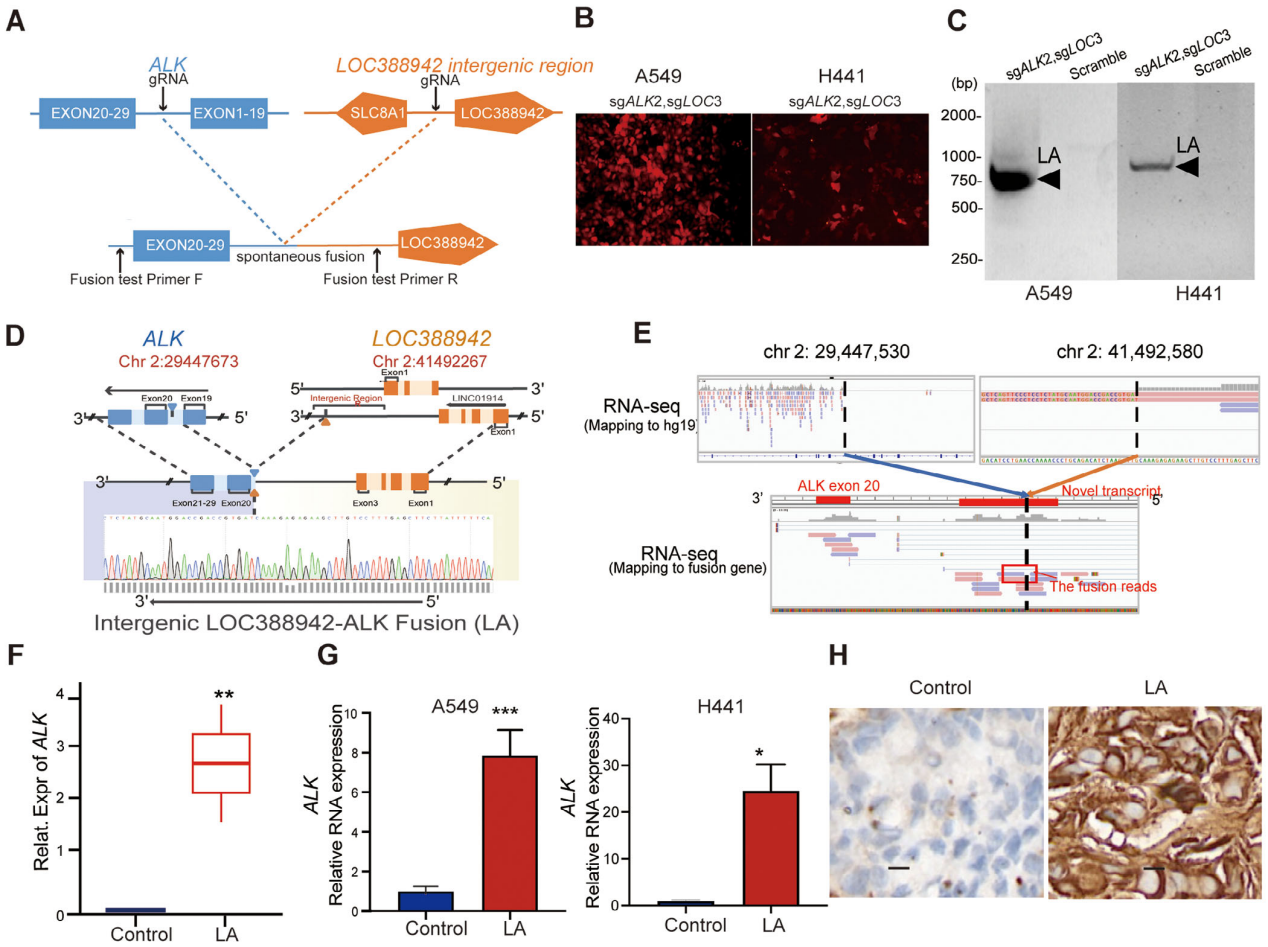
Construction of spontaneous fusion of *LOC388942‐ALK* in A549 and H441 cells. (A) Schematic layout showing the experimental design of *LA* spontaneous fusion. (B) Fluorescence representative images of A549‐Cas9 and H441‐Cas9 cells infected by lentivirus V2Tc containing sg*ALK*2 and sg*LOC388942*. (C) Fusion‐specific PCR of CRISPR/Cas9 gene editing A549 cells and H441 cells. (D) Schematic representation and Sanger sequencing of the *LOC388942‐ALK* fusion. The yellow blocks represent exons of the *LOC388942* gene, and the blue blocks represent exons of the *ALK* gene. The Sanger sequencing at the breakpoint site is shown at the bottom. Chromosome breakage loci were chr 2:29447673 and chr 2:41492267, respectively. (E) Identifying *LA* fusion transcripts using RNA‐seq presenting using Integrated Genomics Viewer (IGV). The IGV based on RNA‐seq displays the transcribed reads in the *LA* fusion. (F) Boxplot showing the relative expression levels of *ALK* in control and tumor cells with *LA*. **p<0.01;(G) qPCR analysis of ALK expression in A549 and H441 cells with *LA* fusion; mean ± standard deviation (SD); *n* = 6; ‐ *p < 0.05,***p<0.001; Wald test. Boxplots show the IQR divided by the median. Whiskers represent the minimum and maximum values at 1.5^∗^IQRs. (H) Representative immunohistochemical staining showing high ALK protein expression in cells with *LA* fusion (original magnification 200×).

To explore the functional roles of the intergenic *ALK* fusion—*LOC388942‐ALK*—in NSCLC, spontaneous fusion was accomplished by designing sgRNAs between exons 19 and 20 of *ALK* as well as between *LOC388942* and *SLC8A1* both in A549 and H441 cell lines, which was validated by fluorescence and PCR assay (Figure 2A–C). DNA sequencing showed that breakpoint sites of our constructed *ALK* fusion were chr 2:29447673 and chr 2:41492267 in A549 cell line, respectively, which are similar to previous *ALK* fusion breakpoint sites in patient samples (Figure 2D), while RNA sequencing showed the intergenic region transcript in RNA (Figure 2E). Further, RNA‐seq, qPCR assay, and IHC staining also demonstrated the presence of RNA expressing *LOC388942‐ALK* in our constructed fusion cells (Figure 2F–H). In summary, we successfully constructed *LOC388942‐ALK* fusion in A549 cells and H441 cells, which is similar to that of patient samples.

### 2.3 *LA* Fusions Have Oncogenic Effects

**FIGURE 3:**
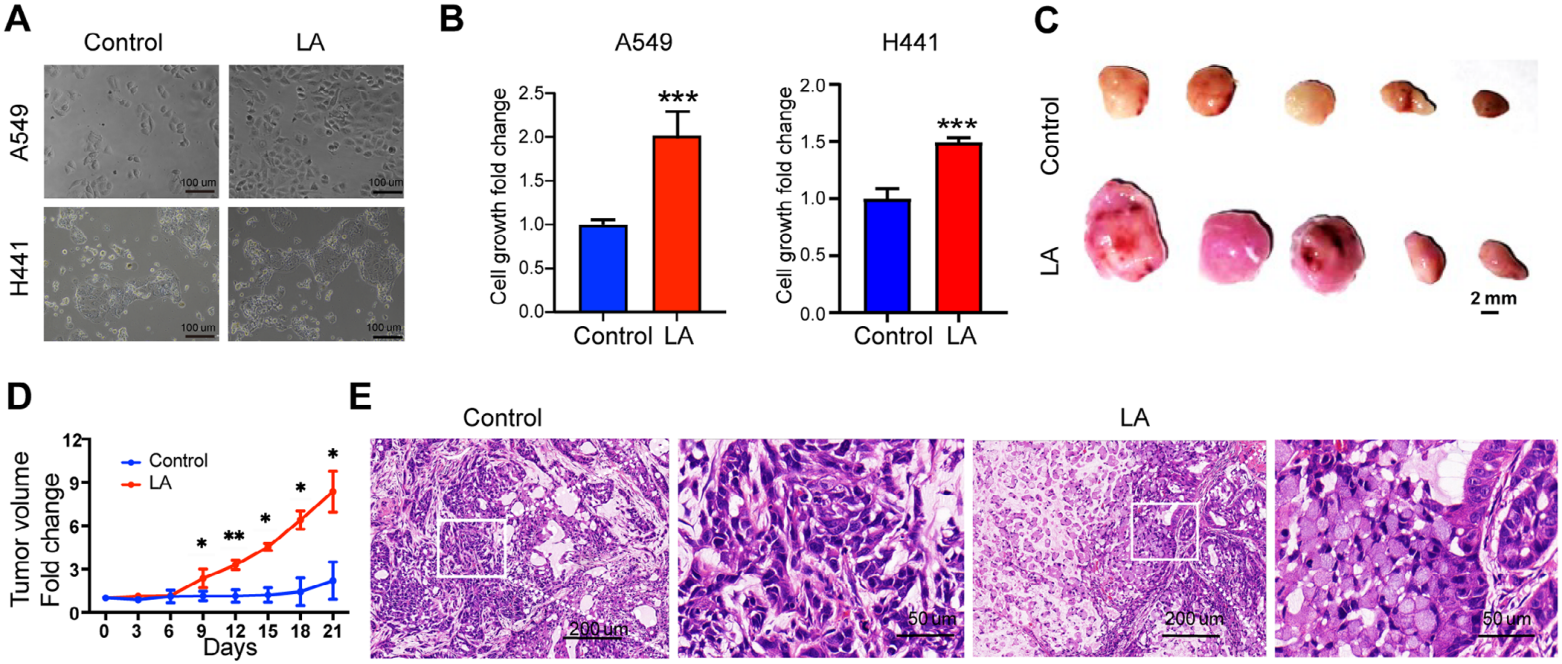
The oncogenic role of *LA* fusion in NSCLC. (A) Bright‐field images of A549 cells and H441 cells with or without *LA* after cultured for 24 h; scale bars, 100 µm. (B) The bar graph shows the fold change of cell growth of A549 cells and H441 cells with or without *LA*; ***p < 0.001; two‐sided Student's *t*‐test; data presented as mean ± SD. (C) Bright‐field image of tumors with or without *LA* (*n* = 5 mice); scale bars, 2 mm. (D) The tumor volume fold change of A549 tumors with or without *LA* (*n* = 5 mice); ^∗^ *p* < 0.05, ^∗∗^ *p* < 0.01; two‐sided Student's *t*‐test; data presented as mean ± SD. (E) Representative H&E stainings of tumors with or without *LA* (representative of *n* = 3 mice); scale bars, 200 and 50 µm.

*EML4‐ALK* fusion is a classic driver gene in NSCLC. To investigate whether tumors with *LA* fusion also have oncogenic effects, we cultured control cells without *LA* fusion and tumor cells with *LA* fusion in vitro. We found that tumor cells with *LA* fusion grew significantly faster than the control group (Figure 3A,B). We then transplanted these two types of cells subcutaneously into nude mice, and the consistent result was that tumors with *LA* fusion grew significantly faster than control tumors (Figure 3C,D). Pathological analysis showed that compared with the control group, tumors with *LA* fusion had a significantly increased number of signet ring‐like cells in hematoxylin and eosin (H&E) staining (Figure 3E), indicating that tumors with *LA* fusion have the typical pathological characteristics of tumors with ALK fusion.

### 2.4 Tumors With *LA* Fusion Were Resistant to Alectinib

**FIGURE 4:**
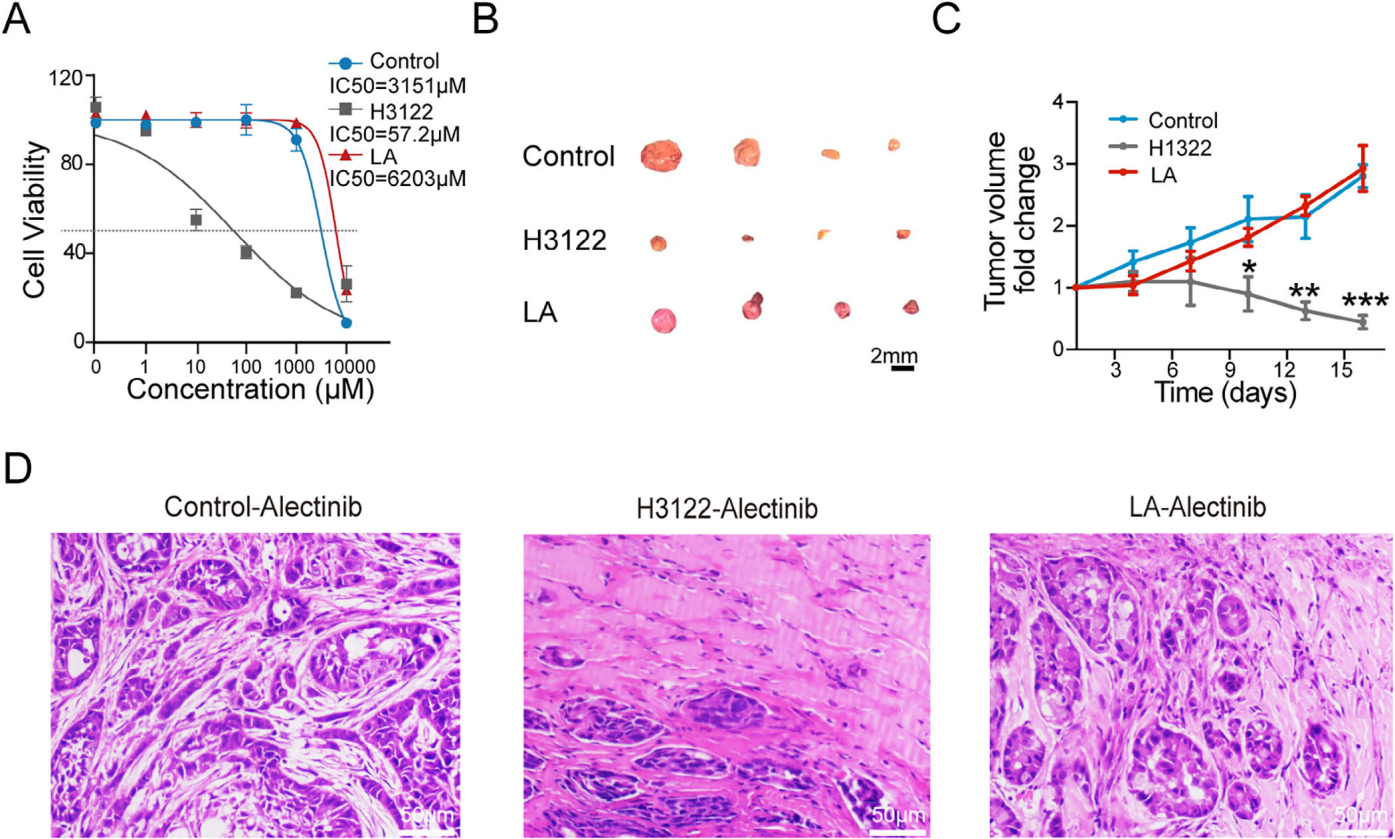
Tumors with *LA* fusion treated with alectinib. (A) Dose‐response curves of control, H1322, or *LA* tumor cells treated with alectinib (*n* = 3 technical replicates); **p < 0.01, ***p < 0.001; two‐sided Student's *t*‐test; data presented as mean ± SD. (B) Bright‐field image of control, H1322, and *LA* tumors treated with alectinib; scale bars, 2 mm. (C) The curves showing the fold change of tumor volumes of control, H1322, or *LA* tumor cells treated with alectinib (*n* = 4 mice); *p < 0.05, **p < 0.01, ***p < 0.001; two‐sided Student's *t*‐test; data presented as mean ± SD. (D) Representative H&E staining images of control, H1322, or *LA* tumors treated with alectinib (*n* = 4 mice per group).

Alectinib is a second‐generation ALK‐TKI administered to NSCLC patients with the *EML4‐ALK* fusion gene [19]. We wondered whether tumors with *LA* fusion were sensitive to alectinib. We treated control, H1322, and *LA* tumor cells with alectinib in vitro and found that tumor cells with *LA* fusion were significantly more resistant than H1322 cells with *EML4‐ALK* fusion (Figure 4A). We then tested the in vivo treatment efficacy of alectinib in control, H1322, and *LA* tumors. Consistently, alectinib treatment significantly repressed the growth of H1322 tumors, while it had no significant effect on the growth of the control and *LA* tumors (Figure 4B,C). Pathologic analyses revealed that the H1322 tumors treated with alectinib displayed large areas of necroptotic cells. In contrast, more survival tumor cells were observed in control and *LA* tumors (Figure 4D), indicating that tumors with *LA* fusion were resistant to ALK‐TKI.

### 2.5 Tumors With *LA* Fusion Were Marked by Proliferative Tumor Cells and a Tendency for Invasion and Metastasis

**FIGURE 5:**
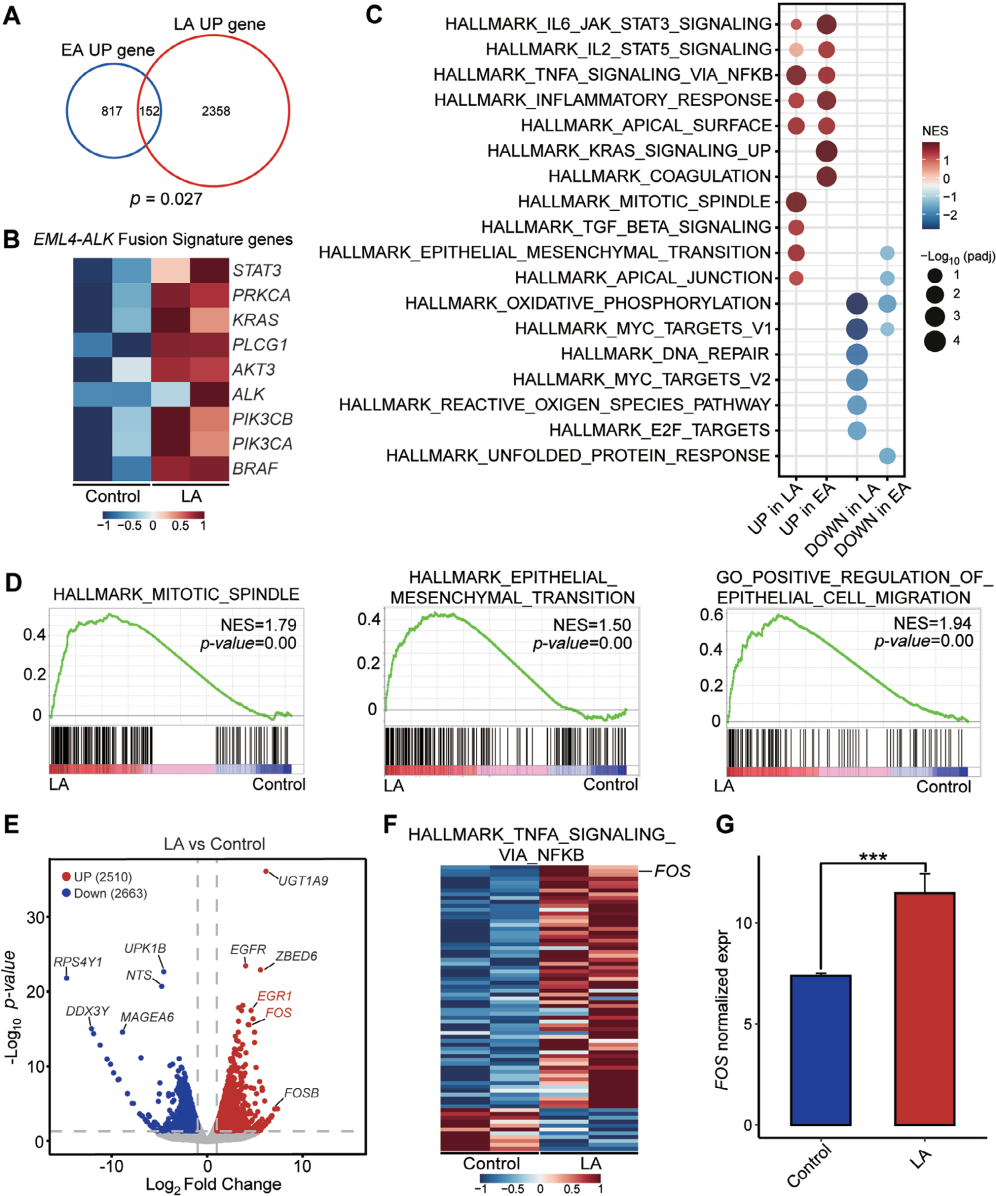
The transcriptomic analysis of *LA* intergenic fusion. (A) The Venn diagram showing the overlap of upregulated genes in *EML4‐ALK* cells compared to vector and in *LA* cells compared to control (hypergeometric test). (B) Heatmaps showing upregulated *EML4‐ALK* fusion signature genes in tumor cells with or without *LA* fusion. (C) Bubble plot based on GSEA of differentially expressed pathways of downregulated pathways (blue) and upregulated pathways (red) in *LA* cells and *EML4‐ALK* cells. (D) GSEA showing the positive enrichment of the HALLMARK_MITOTIC_SPINDLE gene set, the HALLMARK_EPITHELIAL_MESENCHYMAL_TRANSITION gene set, and the GO_POSITIVE_REGULATION_OF_EPITHELIAL_CELL_MIGRATION gene set in *LA* cells compared to control. (E) Volcano plot showing differentially expressed genes in *LA* cells and controls. Significantly upregulated genes (red) and downregulated genes (blue) were selected with the absolute value of fold change *p* < 0.5 and *p* < 0.05. (F) Heatmap showing the HALLMARK_TNFA_SIGNALING_VIA_NFKB genes in tumor cells with or without *LA* fusion. (G) mRNA expression of *FOS* in tumor cells with or without *LA* fusion; ***p < 0.001; Wald test.

We performed RNA‐seq in duplicated samples of A549 cells with and without *LA* intergenic fusion. We found that the significantly upregulated genes in *LA* fusion partially overlap with those in *EML4‐ALK* (*EA*) fusion (Figure 5A). In addition, *LA* fusion cells exhibited high expression of certain *EA* signature genes, including *ALK*, *STAT3*, and *KRAS* (Figure 5B). Further, the transcriptomic similarity between the *LA* fusion and the *EA* fusion was revealed by gene set enrichment analysis (GSEA) with upregulated gene signatures of some classic *EA* fusion pathways, such as JAK/STAT3 signaling pathway (Figure 5C). These data suggested that *LA* fusion shares some characteristics of *EA* fusion. However, the results of GSEA also identified the mitotic spindle pathway as significantly upregulated by *LA* expression in A549, along with some pathways related to tumor migration involving epithelial–mesenchymal transition and epithelial cell migration (Figure 5D). It is marked by proliferative tumor cells and a tendency for invasion and metastasis. We searched for candidate genes that *LA* specifically upregulated. *FOS* gene was at the top of the list (Figure 5E). Additionally, the HALLMARK_TNFA_SIGNALING_VIA_NFKB gene set with high *FOS* expression was significantly positively enriched in *LA* cells compared to control cells (Figure 5F,G). The results showed that the *FOS* gene is a potential therapeutic target for *LA*.

### 2.6 Targeting *FOS* as a Potential Treatment Option for Tumors With *LA* Fusion

**FIGURE 6:**
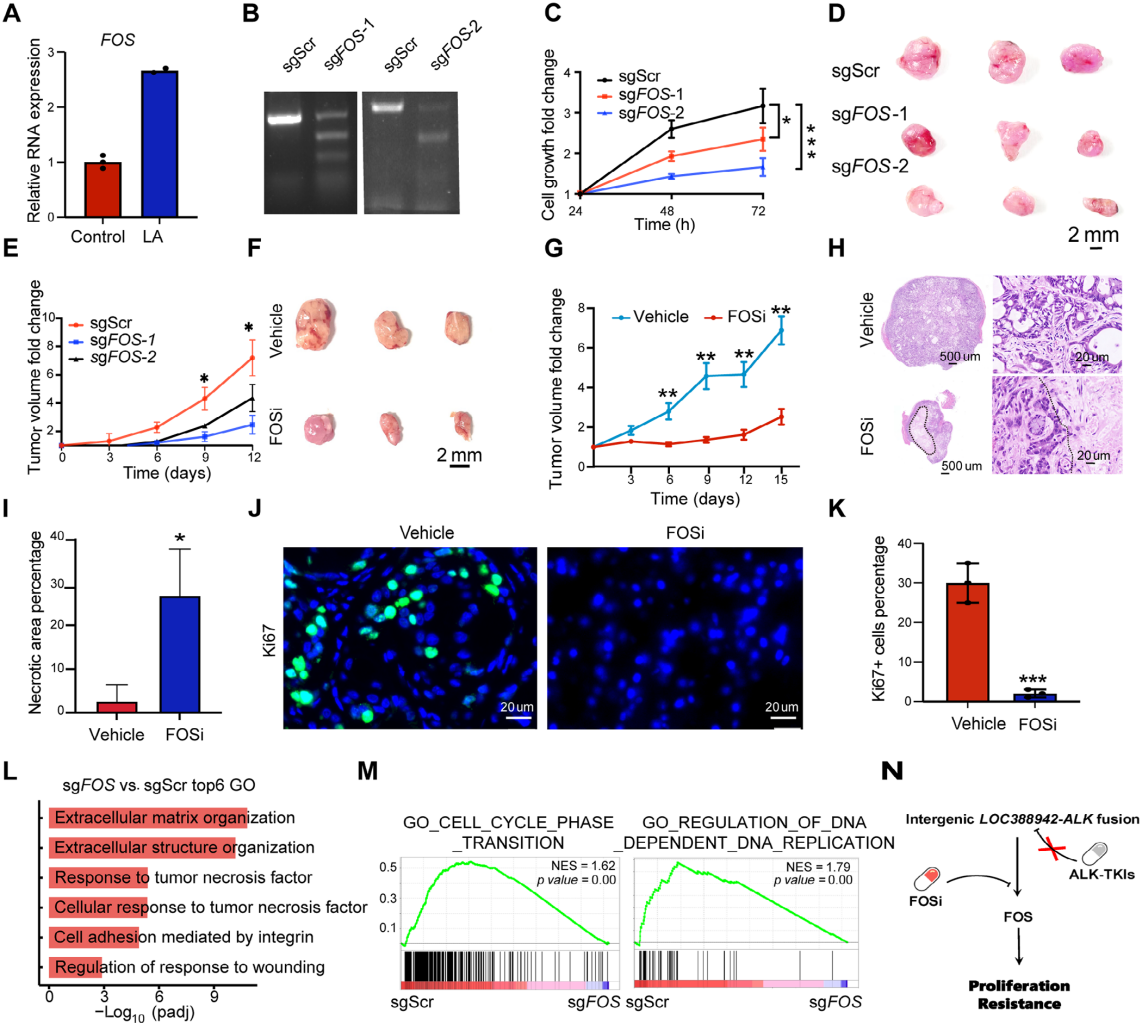
The role of *FOS* in A549 cell line with LOC388942‐ALK fusion. (A) The relative mRNA levels of *FOS* of A549 cells with or without *LA* fusion (*n* = 3 technical replicates); data presented as mean ± SD. (B) 7E1 assay of sgScr, sg*FOS*‐1, and sg*FOS*‐2 of A549 cell line with LOC388942‐ALK fusion. (C) The tumor cell growth fold change of sgScr, sg*FOS*‐1, and sg*FOS*‐2 of the A549 cell line with LOC388942‐ALK fusion (*n* = 3); ∗*p* < 0.05, ∗∗∗*p* < 0.001; two‐sided Student's *t*‐test; data presented as mean ± SD. (D) Bright‐field image of sgScr, sg*FOS*‐1, and sg*FOS*‐2 tumor of A549 cell line with LOC388942‐ALK fusion; scale bars, 2 mm (*n* = 3 mice per group). (E) Curves showing the tumor volume fold change of sgScr, sg*FOS*‐1, and sg*FOS*‐2 of A549 cell line with LOC388942‐ALK fusion (*n* = 3 mice per group). *p<0.05;(F) Bright‐field image of the *LA* tumors treated with vehicle and FOS inhibitor; scale bars, 2 mm (n = 3 mice per group). (G) Curves showing the tumor volume fold change of the *LA* tumors treated with vehicle and FOS inhibitor (*n* = 3 mice per group). **p<0.01; (H) Representative H&E staining images of the *LA* tumors treated with vehicle and FOS inhibitor (*n* = 3 mice per group). (I) Percentage analysis of necrosis area of the *LA* tumors treated with vehicle and FOS inhibitor (*n* = 3 mice per group). *p<0.05; (J) Representative Ki67 staining images of *LA* tumors treated with vehicle and FOS inhibitor (*n* = 3 mice per group); scale bars, 20 µm. (K) The bar graph shows the Ki67 positive cell percentage of *LA* tumors treated with vehicle and FOS inhibitor (*n* = 3 mice per group). ***p<0.001; (L) Bar plot showing the positive enrichment of pathways when compared sgScr to sg*FOS* in A549 cell line with LOC388942‐ALK fusion. (M) GSEA showing the positive enrichment of gene set when compared sgScr to sg*FOS* in A549 cell line with *LOC388942‐ALK* fusion, (N) Schematic diagram of the mechanism of *LOC388942‐ALK* fusion.

To investigate the function of the *FOS* gene, qPCR showed that *FOS* was highly expressed in *LA* tumors (Figure 6A). We performed *FOS* gene knockout in A549 cells with *LA* fusion (Figure 6B). The tumor cell growth was significantly reduced in vitro and in vivo following *FOS* knockout (Figure 6C–E). Since tumors with *LA* were resistant to ALK‐TKI, we wondered whether there is a potential drug to treat *LA* tumors. We proposed that T5224, an inhibitor of FOS, might be an effective drug for tumor cells with *LA*. We found that intraperitoneal injection of T5224 significantly restrained the growth of *LA* tumors in mice (Figure 6F,G). The histological assay showed that there was a significantly increased area of necroptotic cells in the T5224‐treated tumors than in the vehicle‐treated ones (Figure 6H,I). The cell proliferation was significantly inhibited in the T‐5224–treated tumors, as indicated by Ki67 positive cell percentages (Figure 6J,K). We found that Response_to_tumor_necrosis_factor and Cellular_response_to_tumor_necrosis_factor pathways were enriched, while GO_CELL_CYCLE_PHASE_TRANSITION and GO_REGULATION_OF_DNA_DEPENDENT_DNA_REPLICATION were decreased in sg*FOS* of A549 cell line with LOC388942‐ALK fusion when compared to sgScr (Figure 6L,M). Thus, T‐5224 could be an effective drug for tumors with *LA* fusion by inhibiting *FOS*.

## 3 Discussion

Intergenic breakpoint fusions, where one or both genomic breakpoints localize to intergenic regions, are theoretically unlikely to be functional due to the lack of chimeric full‐coding transcripts and, consequently, the absence of chimeric fusion proteins [20]. However, our study found that at the RNA level, the intergenic region of *LOC388942* was transcribed, and chimeric fusion proteins were formed with *ALK*. These observations align with a previous study, which identified an intergenic‐3′*ROS1* fusion with a breakpoint downstream of tropomyosin 3 (*TPM3*), resulting in positive ROS1 expression and transcription involving exon 8 of *TPM3*. Alternative splicing mechanisms may contribute to in‐frame fusion transcripts by skipping the stop codon of *TPM3* [21]. Furthermore, a study suggested that intergenic fusions can skip the first exon of the 3′ gene or alter the position or composition of gene regulatory elements, leading to overexpression of downstream genes [13]. These findings are significant as they offer new insights into how partial intergenic breakpoint fusions could achieve transcriptional activity.

Upon confirming the transcription and expression of intergenic fusions, we further investigated the differences in oncogenic effects between *LOC388942‐ALK* and the classic *EML4‐ALK*. Our findings showed that *LA* fusion cells exhibited a pronounced proliferation advantage both in vitro and in vivo, consistent with the known role of ALK fusions in promoting tumorigenesis [22]. Pathological analyses confirmed that *LA* fusion tumors displayed typical features associated with *ALK f*usions, reinforcing that *LA* fusion is a novel oncogenic driver in NSCLC. Importantly, *LA* fusion cells demonstrated significant resistance to the ALK inhibitor, alectinib, unlike *EML4‐ALK* fusion cells, suggesting that distinct molecular pathways regulate the survival of *LA* fusion cells in response to therapy. Drug resistance in this context is often linked to gene mutations, bypass activation, or pathological transformation [7, 23]. The upregulation of compensatory survival pathways, specifically the HALLMARK_TNFA_SIGNALING_VIA_NFKB gene set involving high levels of *FOS* in *LA* fusion tumor cells, may explain this resistance. This resistance profile provides insights into the heterogeneity of ALK‐driven NSCLC and highlights the need for developing therapeutic agents specifically designed to counteract such unique mechanisms.

The relationship between the *LA* fusion and the *FOS* gene is crucial for understanding the molecular mechanisms underlying the oncogenic properties of *LA* fusion in NSCLC. Our findings indicated a significant upregulation of the *FOS* gene in *LA* fusion cells, suggesting its role as a mediator of the fusion's oncogenic effects. As a member of the AP‐1 transcription factor family, *FOS* is involved in various cellular processes, including proliferation, differentiation, and apoptosis, and its dysregulation is associated with several malignancies, making it a promising target for therapeutic intervention [24, 25, 26, 27]. The upregulation of *FOS* in *LA* fusion cells may contribute to their enhanced proliferative capacity, as evidenced by the efficacy of the FOS inhibitor T5224 in suppressing *LA* fusion cell proliferation in both in vitro and in vivo models. Therefore, our study implies that *FOS* upregulation is closely associated with the oncogenic effect of *LA* fusion, identifying it as a viable therapeutic target.

In *ALK* fusion‐positive NSCLC, novel variant *ALK* fusions (e.g., non–*EML4‐ALK* fusions) are increasingly being identified alongside the canonical *EML4‐ALK* fusion [10]. However, the oncogenic mechanisms of many variant *ALK* fusions remain poorly characterized, and targeted therapies for these variants are lacking. Clinically, physicians often empirically administer ALK‐TKIs developed for *EML4‐ALK*, such as crizotinib or alectinib, to patients harboring variant *ALK* fusions. Studies demonstrate that non–EML4‐ALK fusions treated with conventional ALK‐TKIs exhibit significantly poorer clinical outcomes [28, 29]. To address this unmet need, our proposed strategy—generating patient‐matched variant *ALK* fusion models (both in vitro and in vivo), systematically investigating their oncogenic mechanisms, biological functions, and tumor‐driving molecular pathways, and identifying effective therapeutic interventions—could help standardize and optimize treatment paradigms for patients with tumors driven by variant *ALK* fusions.

## 4 Materials and Methods

### 4.1 Sex as a Biological Variable

We found *LOC388942‐ALK* in a male patient. Our study exclusively examined male mice and male human specimens. It is unknown whether the findings are relevant for female mice.

### 4.2 Sample and Patients

Our study collected a total of 34,070 NSCLC cases from January 2010 to October 2021 at the Department of Pathology, West China Hospital, Sichuan University, who had undergone at least one of the ALK‐V, FISH, RT‐PCR, and NGS tests. These cases were compiled into a comprehensive *ALK* database. From this database, a total of 378 patients who tested positive for all four ALK examinations were identified. This study was approved by the Biomedical Ethics Committee of West China Hospital, Sichuan University (Approval No. 2023–987 and 2024–147).

### 4.3 Mice

Mice were kept in a pathogen‐free animal facility at Sichuan University, where food, bedding, and water were autoclaved. All animal procedures were followed by ARRIVE guidelines and the National Research Council's Guide for the Care and Use of Laboratory Animals and approved by the Animal Care and Use Committee of Sichuan University (No. 20181204027). BALB/cA‐nu mice (Beijing HFK Bioscience, Cat# 13001A) (male, 6–8 weeks, and ∼20 g weight) were purchased for our study. The tumor volume of mice was monitored by bioluminescent imaging.

### 4.4 Cell Culture

HEK 293T cells (CRL‐1573) were purchased from ATCC and cultured at 37°C with 5% CO_2_ in DMEM supplemented with 10% (vol/vol) fetal bovine serum (WISENT, Cat# 086‐150) and penicillin (100 U mL^−1^)/streptomycin (0.1 mg mL^−1^). At the same time, A549 cells(CL‐0016) and H441cells (CL‐0514) were from Procell and cultured at 37°C with 5% CO_2_ in RPMI medium1640 basic supplemented with 10% (vol/vol) fetal bovine serum (WISENT, Cat# 086‐150) and penicillin (100 U mL^−1^)/streptomycin (0.1 mg mL^−1^). The HEK 293T, A549 cell, and H441 cell lines were routinely tested for *Mycoplasma* by PCR. Experiments were performed within 4 weeks after fresh viable cells were thawed.

### 4.5 H&E, IHC, and Immunofluorescence (IF)

The tumor sections, with a thickness of 5 µm, were cut from formalin‐fixed paraffin‐embedded samples. To deparaffinize the sections, the sections were immersed in xylene for 5 min, repeating this step thrice. Then, proceed with rehydration by sequentially exposing the sections to decreasing ethanol concentrations (100%, 95%, and 70% ethanol) and, finally, distilled water. After that, H&E staining was performed following the standard protocol. Regarding IHC and IF staining, primary antibodies were applied at 1:50–1:500 dilution in 2% goat serum and incubated overnight at 4°C. Primary antibodies contained ALK (Cell Signaling Technology, Rabbit mAb, # 3633) and Ki67 (HUABIO, Rabbit mAb, # ER1706‐46). For nuclear staining, a two‐step detection kit (PV‐9001 and PV‐9002) was used for IHC and hematoxylin. As for IF, fluorescence‐conjugated secondary antibodies were used.

### 4.6 Fluorescence In Situ Hybridization

FISH evaluation for ALK gene rearrangement was performed on the 5 µm lung cancer tissue sections using the ALK dual color, break‐apart rearrangement probe (Anbiping, Guangzhou, China). The probe contains two differently labeled probes on opposite sides of the breakpoint of the ALK gene. A probe approximately 250 kb for the telomeric side of the ALK breakpoint is labeled with SpectrumOrange, and the centromeric probe is approximately 300 kb and labeled with SpectrumGreen. The results were scored in 200 nonoverlapping nuclei, and positivity was defined as > 15% split signals in tumor cells.

### 4.7 Plasmid and Intergenic ALK Fusion Cell Models Construction

Based on the sequencing data of the patient samples, the CRISPR sgRNA design tool was used to import the sequences of the intergenic region of the *LOC388942* gene and the sequences of 150 bp upstream and downstream of the breakpoint of the *ALK* gene, respectively, to design and synthesize the corresponding sgRNAs and to add sticky ends at both ends of the oligo (Table S1). sgRNAs mentioned above were annealed and ligated into the BsmBI‐digested lentiviral viral vector (pLentiCRISPRV2‐U6‐EFS‐mCherry, V2Tc) and coated plates. V2Tc‐sg*LOC388942* plasmid and V2Tc‐sg*ALK* plasmid were extracted for enzymatic characterization, respectively. Lentiviruses with the above two sgRNAs were packaged in 293T cells using calcium phosphate transfection. The viruses were collected at 36, 48, and 72 h, and then used to infect A549‐Cas9 and H441‐Cas9 cells, which lack *ALK* fusions but have inactivated *TP53*. The cells were cultured for 2 days, and infection efficiency was assessed by fluorescence microscopy and T7EI digestion. Cells sequenced as described above containing *LOC388942*‐*ALK* (*LA*) intergenic fusions were counted using the doubling dilution method, which ultimately ensured that there was one cell per 100 µL of culture medium. After 2–3 weeks of culture, cells formed monoclonal clusters, the whole genome was extracted, and the *LA* intergenic fusion was amplified using fusion PCR. Finally, Sanger sequencing and RNA sequencing verified the spontaneous fusion production of each monoclonal cell strain.

### 4.8 PCR for Detecting Fusion

F and R primers were designed 500 bp upstream of the 5′ end of the sg*LOC388942* sequence and 500 bp downstream of the 3′ end of the sg*ALK* sequence and were produced by Tsingke, respectively. The primer sequences are listed in Table S2. The PCR products of 500–1000 bp amplified by the above two primers were then considered to be the spontaneous fusion of *LA*. After adding 100 ng genome to phanta enzyme (Tsingke, Beijing, China), PCR primers, and water, setup the program at 95°C for 3 min, totaling one cycle; 95°C for 15 s, 58°C for 15 s, and 72°C for 1 min, totaling 35 cycles; and 72°C for 5 min, totaling one cycle and then held at 16°C. A total of 3 µL product was added to a 3% agarose gel, electrophoresed at 150 V for 15 min, and then placed into a gel imaging analysis system (Flour‐S MultiImager, BIO‐RAD) for imaging.

### 4.9 RNA Extraction and qPCR

Total RNA extraction used TRIzol reagent (Applied Biosystems, Cat# 15596026) according to the instructions. Hiscript III RT SuperMix for qPCR (+gRNA wiper) (Vazyme, R323‐01) was used for complementary DNA (cDNA) synthesis. RT‐qPCR was conducted on a QuantStudio 3 platform (Applied Biosystems) using PowerUp SYBR Green master mix (Applied Biosystems, A25741). The relative expression of genes was determined using the 2^−ΔΔ^ *^Ct^* method. Gene expression levels were normalized to *Actin*. Each sample was subjected to triplicate RT‐qPCR runs. Primer sequences are listed in Table S3.

### 4.10 RNA‐Sequence

RNA‐seq libraries were constructed using Illumina Stranded mRNA Sample Preparation Kit (NEB, Cat# E7770) according to the manufacturer's protocol and were sequenced by Illumina NovaSeq 6000 sequencing machine with 150‐bp paired‐end reads.

The RNA‐seq reads were aligned to the *Homo sapiens* reference genome (hg19) by STAR. DESeq2 (v1.26.0) was used to identify differential expression genes. The differentially expressed mRNAs were selected with the absolute value of |log_2_fold‐change| > 0.5 and *p* < 0.05. Pheatmap (v1.0.12) was used to display heatmaps of the expression levels of differentially expressed genes, which were normalized by *z*‐score. GSEA was utilized to identify significantly enriched pathways using default parameters. RNA sequencing data of the EML4‐ALK fusion was downloaded from the Gene Expression Omnibus (GEO) database with the accession number, GSE165137.

### 4.11 Cell Growth In Vitro

Cell viability was verified with a cell counting kit‐8 (CCK8; MCE, America). Tumor cells with or without *LA* fusion were seeded into the 96‐well plate at a density of 5 × 10^3^ cells per well. After 24 h of incubation, 100 µL of medium containing 10% reagent was added to each well before the analysis. After incubation at 37°C for 1 h, the absorbance values at OD 490 nm were measured using a microplate reader (Molecular Devices, USA) to calculate the viability ratio.

### 4.12 Tumor Growth in Vivo

Tumor cells with or without *LA* were digested with trypsin at 37°C for 5 min and spun at 400 × *g* for 5 min at room temperature. The collected cells were resuspended with 50% Matrigel mixed with PBS. Cell suspension was injected under the skin of BALB/c nude mice (male, 6–8 weeks). Each mouse had two subcutaneously injected tumor sites with 1 × 10^6^ cells per site. Tumor volumes were measured every 3 days by caliper. Mice were sacrificed, and pathology was analyzed at the indicated time points.

### 4.13 Drug Treatments

For in vitro treatment, alectinib (MCE, Cat# HY‐13011) or FOS inhibitor (FOSi) (Selleck, Cat# S8966) was added at the indicated concentrations into three replicate wells with control, H3122 and *LA* tumor cells. Cell viability was measured at 72 h after treatment. For in vivo treatment, approximately 1 × 10^6^ control and *LA* tumor cells were subcutaneously transplanted into 6‐week‐old nude mice. Mice were monitored for tumor burden by caliper every 3 days. Alectinib and FOSi treatment was initiated when tumor volume reached about 100 mm^3^. Mice were allocated into two groups for vehicle or FOSi treatment every day. Mice were killed and analyzed once moribund or at the indicated time points.

### 4.14 Statistics

All in vitro and in vivo experiments were analyzed using GraphPad Prism (version 9, RRID: SCR_002798), with quantitative data assessed by a two‐tailed Student's *t*‐test. The number of independent experiments, samples, or events is detailed in the figure legends. Unless otherwise noted, data are presented as mean ± standard deviation (SD). For in vitro treatments, samples were randomly assigned to vehicle or treatment groups, and blinding was used for tumor measurements and cell viability analysis. In vivo, treatment groups were randomized based on tumor burden before treatment. No data were excluded from the analysis. Statistical bioinformatics methods are described in the figure legends. R version 3.6 was used for omics data analysis and visualization, with “ggplot2” for graph generation. Statistical significance for Venn plots was assessed using a hypergeometric test. Results were considered significant when *p* < 0.05 (*p < 0.05, **p < 0.01, ***p < 0.001).

### 4.15 Study Approval

This study adhered to the Helsinki Declaration of the World Medical Association. The Biomedical Ethics Committee of West China Hospital, Sichuan University officially approved the study, which can be extracted from the Chinese Clinical Trials Registry (ChiCTR2100052715). Informed written consent was obtained from each patient before enrollment. The clinicopathological information was collected, and then patient identifiers were removed.

The animal experiments were approved by the West China Animal Ethics Committee (20240301070).

## Author Contributions

X Z, M W, Q Z, Y L, C C: conceived the project and designed the experiments; X Z, M W, Q Z, YM W, DL L: performed the experiments, analyzed the data; ZY L, WY W, JW L, GW C, QH Z, Y L, C C: provide resource; X Z, M W, Q Z, YM W, DL L:write the original manuscript; X Z, M W, Q Z, YM W, DL L, JW L, GW C, QH Z, Y L, C C: edit and review the manuscript. The order of cofirst authors was determined by the relative amount of data each contributed. All authors have read and approved the final manuscript.

## Ethics Statement

This study adhered to the Helsinki Declaration of the World Medical Association. The Biomedical Ethics Committee of West China Hospital, Sichuan University, officially approved the study (Approval No. 2023–987 and 2024–147), which can be extracted from the Chinese Clinical Trials Registry (ChiCTR2100052715). All animal procedures were approved by the Animal Care and Use Committee of Sichuan University (No. 20181204027).

## Consent

Informed written consent was obtained from each patient before enrollment. The clinicopathological information was collected, then patient identifiers were removed.

## Conflicts of Interest

The authors declare no conflicts of interest.

## Supporting information



Supporting Information

## Data Availability

The RNA‐seq data will be deposited into the Gene Expression Omnibus (GEO) database repository for public access under accession number GSE290536. RNA sequencing data of the EML4‐ALK fusion was downloaded from the GEO database (GSE165137). All data are available in the main text or the Supporting Information.
