# Supplementary material for: Identifying the Intergenic ALK Fusion LOC388942‐ALK as a Driver of Non–Small Cell Lung Cancer
Source: MedComm (2020). 2025 Mar 27;6(4):e70154. doi: 10.1002/mco2.70154 (PMC11949501; doi:10.1002/mco2.70154)
Supplement: Supplementary file 1 — Supporting Information [file MCO2-6-e70154-s001.docx]

**Supplementary table 1**

**Table of gRNA**

| gRNAs | Sequences (5’-3’) |
| --- | --- |
| H-ALK-gRNA-1F | CACCGGACCGACCGTGATCAGATT |
| H-ALK-gRNA-1R | AAACAATCTGATCACGGTCGGTCC |
| H-ALK-gRNA-2F | CACCGTTCAATTCATTCGATCCTC |
| H-ALK-gRNA-2R | AAACGAGGATCGAATGAATTGAAC |
| H-LOC388942-gRNA-3F | CACCGTGCAATCTTAGATGTCTGC |
| H-LOC388942-gRNA-3R | AAACGCAGACATCTAAGATTGCAC |
| H-Fos-gRNA-1F | CACCGTTCGTCTTCACCTACCCCG |
| H-Fos-gRNA-1R | AAACCGGGGTAGGTGAAGACGAAC |
| H-Fos-gRNA-2F | CACCGGGTGACCACCGGAGTGCAC |
| H-Fos-gRNA-2R | AAACGTGCACTCCGGTGGTCACCC |

**Supplementary table 2**

**Table of LOC388942-ALK Fusion-specific PCR of CRISPR-Cas9 gene editing A549 and H441 cell primers used for PCR**

|  | Sequence (5’-3’) |
| --- | --- |
| Forward | CATGTGAGGATGTTCTGGAAGG |
| Reverse | ATCCCATGTCATCCAGTAAACC |

**Supplementary table 3**

**Table of primers used for q-PCR**

| Target |  | Oligo sequence (5’-3’) |
| --- | --- | --- |
| c-Fos | Human | F: CCGGGGATAGCCTCTCTTACT  R: CCAGGTCCGTGCAGAAGTC |
| ALK | Human | F: AATCGGGCGTCCAGACAAC  R: GAGCCTGGGGATGTTCCTTC |
| Actin | Human | F: CATGTACGTTGCTATCCAGGC  R: CTCCTTAATGTCACGCACGAT |
